# Distribution of Blood Vessels in the Lungs

**Published:** 1854-06

**Authors:** James Newton Heale


### *On the distribution of the Bloodvessels in the Lungs.*

BY JAMES NEWTON HEALE, M. D.

The author's investigations have led to some very interesting results, of which the following is a summary:

1. The pulmonary artery makes no anastomoses whatever with any other artery, nor do its own branches anastomose together. Its branches go direct to the air-cells, and are there distributed, and terminate as arteries [*i. e.*, in capillaries]. None of its branches go to any other tissues of the lungs besides the air-cells, except some few which perforate the subpleural cellular [areolar] tissues, and are distributed to the pleura; some of these also cross the posterior mediastinum, beneath the pleura, and reach the thoracic pleura.

2. But the capillaries thus distributed to the air-cells, are extended in a peculiar and very characteristic manner, from the cells to the bronchial mucous membrane; this peculiar vascular plexus being traceable even as high as the trachea, and finally merging in minute venous radicles, which combine to form the pulmonary veins. No branches whatever of the pulmonary artery are distributed to the bronchial membrane, without previously becoming capillary upon and among the air-cells; and no arterial blood is obtained by this membrane from any other source.

3. The bronchial (so called) arteries have their own special distribution. They do not supply any portion of the bronchial mucous membrane; and do not at all communicate with either the pulmonary arteries or veins, except as supplying their cellular sheaths, and therefore, in all probability, furnishing their *vasa vasorum.*

4. The bronchial arteries (injected by filling the *aorta*) terminate in veins, ramifying in the subpleural cellular tissue; and which mostly, after extending on the surface of the lung beneath the pleura, pass between the two layers of pleura forming the ligamentum pulmonis, and finally terminate in the œsophageal and other veins in the posterior mediastinum. Some of them, also, probably terminate in the azygos veins, the jugulars, the diaphragmatic, and the venæ cavæ; in short, wherever they can meet a systemic vein conveniently situated. But these veins form no communication with the pulmonary veins, either in their capillaries or their larger trunks.

5. The pulmonary veins return all the blood carried to the lungs by the pulmonary arteries. They are, therefore, formed from two distinct sets of venous radicles—the first collecting the blood from the perimeters of the air-cells (*i. e.*, the part of the cells farthest from the bronchial tube connected with them,) and from the surface of the lungs and the interlobular spaces; and the second, commencing from the basis of the air-cells, and from the vascular plexus of the bronchial membrane, before described. The larger branches formed by the junction of these two sets, at length accompany the larger bronchi and the pulmonary arteries, and finally terminate in the left auricle of the heart. The blood from the plexus of the bronchial membrane must be different in character from that derived from the air-cells directly, since the epithelium and the bronchial mucus have been derived from it after leaving the air-cells [while the latter is pure and perfectly ærated blood].

6. It is possible to completely inject the pulmonary artery and veins, without at all injecting the bronchial artery or veins. It is also possible thoroughly to inject the latter without at all injecting the former; and in the latter case, the bronchial membrane will remain wholly uninjected, however perfectly the so-called bronchial vessels may have been filled.

7. By injecting the lung through the pulmonary veins, the bronchial membrane becomes thoroughly injected, even before the air-cells are so. But when the pulmonary artery alone is injected, the air-cells become injected long before the liquid reaches the bronchial membrane. In neither of these cases, however, are the so-called brouchial arteries, or the veins corresponping to them, in the slightest degree injected.

8. The coats of the lymphatic vessels of the lung are also supplied by the capillaries directly distributed to the air-cells, and terminating in the pulmonary veins; and vascular plexus upon them strongly resembles that upon the bronchial mucous membrane.—*Annals of Science.*

